# Photochromic and fluorescence properties of coumarin fulgimides

**DOI:** 10.3906/kim-2003-31

**Published:** 2020-08-18

**Authors:** Khamis Nassor ALLY, Leyla ÖZTÜRK, Mahmut KÖSE

**Affiliations:** 1 Department of Chemistry, Faculty of Arts and Science, Zonguldak Bülent Ecevit University, Zonguldak Turkey

**Keywords:** Photochromism, fulgide, fulgimide, coumarin, fluorescence switch

## Abstract

Four new fulgimides possessing a fluorescent coumarin unit were synthesized from the corresponding fulgides, and their photochromic as well as fluorescence properties were investigated. The open-ring forms of coumarin fulgimides were found to exhibit fluorescence in the visible region. Upon exposure to UV light, the fulgimides were transformed into the nonfluorescent closed-ring forms, which can be reverted to the initial fluorescent open-ring forms on exposure to visible light. The efficiency of quenching of fluorescence was as high as 95% at the photostationary state of UV irradiation.

## 1. Introduction

Photochromic compounds, which can be reversibly converted between two isomeric forms by the irradiation of different wavelengths of light, gained great attention in recent years due to their possible industrial applications [1]. Although a number of photochromic systems were reported so far, fulgides and fulgimides [2,3] and the well-known diarylethenes [4–6], all of which show the thermally irreversible 6p-electocyclizations, are the most studied and the most promising photochromic families. Fulgimides have many advantages over the fulgides due to their excellent photochromic behaviors, thermal stabilities, resistivity towards hydrolysis, and versatilities of structural modifications on the nitrogen atom of the imide moiety. To date, many photochromic fulgimides have been synthesized, and their photochromic properties, including thermal stabilities and fatigue resistance, have been investigated [7–9]. However, few fluorescence photoswitchable photochromic fulgimides with the switching ability of fluorescence have been reported so far [10]. The reversible control of fluorescence intensities of a molecule by light is an important issue. In most cases, only one form of a molecule [such as the open form (o-form)] shows a significant fluorescence intensity, while the other form [such as the closed form (c-form)] is not fluorescent. Therefore, the emission intensity of the photochromic molecules can be controlled by the back and forth photoreactions. Such molecular systems attracted much attention due to their possible industrial applications in the optoelectronics area, such as optical memories, bioimaging, and molecular switching devices [11,12].

In 1999, a series of 2-indolylfulgimides with N-alkyl- and N-aryl-imide groups were synthesized by Liang et al. [13]. The c-forms of 2-indolylfulgimides were reported to be fluorescent. In the other papers, Dvornikov et al. reported that a strongly fluorescent benzophenoxazine dye was linked to the photochromic 2-indolylfulgimide to make a composite molecule to use it for the optical memory media. In these papers, the use of photochromic fluorescent molecules in the development of three dimensional (3D) optical memory systems at the terabyte level was discussed in detail [14,15].

Sivasankaran and Palaninathan have reported on a photoswitchable fluorescent polypyrrole film containing 2-indolylfulgimide prepared from 2-indolylfulgimide-functionalized pyrrole monomers by the electropolymerization on an ITO electrode. When the fulgimide part is o-form, the polypyrrole part emitted fluorescent light in the visible region (500–600 nm), while the fulgimide part takes c-form it was not fluorescent due to the Förster resonance energy transfer (FRET) from polypyrrole to the c-form [16].

The coumarins are intrinsically fluorescent [17], and coumarin derivatives are widely used in the organic fluorescent materials because of their strong fluorescent emissions in the visible region. To date, one photochromic fulgimide containing a coumarin moiety as the fluorescent chromophore has been reported. Port et al. synthesized a thienylfulgimide which possesses a 9-anthryl group on the thiophene ring as the photon antenna and a fluorescent coumarin carboxylate group connected to the nitrogen atom of the imide ring by an ethylene linkage. The o-form of the fulgimide displayed an intense emission due to the coumarin group in the visible region, while the photostationary state (pss) of UV-light irradiation showed weak fluorescence due to the remaining o-form [18]. In their study, the photon energy absorbed by the anthryl group migrated through the o-form to the fluorescent coumarin group to give the fluorescent emission, while the photon energy was trapped by the c-form so that the energy transfer to the coumarin moiety did not occur. This was explained by the order of energy of their singlet excited states (anthryl >o-form >coumarin >c-form).

**Figure 1:**
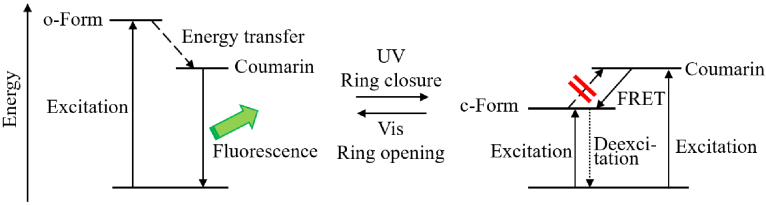
Concept of fluorescence switching of coumarin-containing fulgimides.

**Figure 2:**
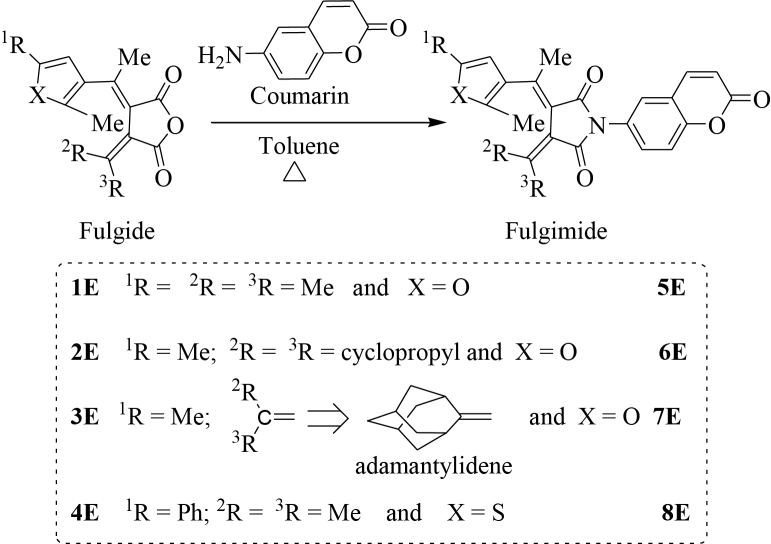
Synthesis of fulgimides.

Our interest was to synthesize and investigate new photoswitchable fluorescent molecules which possess only fulgimide and coumarin. Photochromic reaction changes the energy of the singlet excited state of the fulgimide moiety so that it will control the quenching of the fluorescence of coumarin moiety (Figure 1). To achieve it, the anhydride moiety of fulgides 1E–4E were converted to their fulgimides 5E–8E by the dehydrative condensation of highly florescent 6-aminocoumarin (Figure 2). The coumarin-condensed fulgimides thus prepared were the first examples that the coumarin is attached directly to the imide group of the fulgimides, which were expected to show the fluorescence switching concomitant with the photochromic reactions.

In this study, four new fluorescence-switchable photochromic fulgimides were prepared and their photochromic as well as fluorescence behaviors were examined.

## 2. Material and methods

### 2.1. Material

6-Aminocoumarin hydrochloride was purchased from the Alfa Aesar Company (Haverhill, MA, USA). 1E (^1^R = ^2^R = ^3^R = Me and X = O); 2E (^1^R = Me; ^2^R = ^3^R = cyclopropyl and X = O); 3E (^1^R = Me; (^2^R)(^3^R)C = Adamantylidene, and X = O); and 4E (^1^R = Ph; ^2^R = ^3^R = Me and X = S) were synthesized by a multistep reaction as reported in the literature [19].

### 2.2. Methods

The 1H NMR spectra were measured on a Varian 300 MHz, Agilent 600 MHz NMR spectrometers. Mass spectrum of compounds 6E was measured with AB Sciex 4000 QTRAP LC-MS/MS and mass spectra of compounds 5E, 7E, and 8E were measured with high-performance liquid chromatography/high resolution (timeof-flight) mass spectroscopy (LC-MS/QTOF). FT-IR spectra were measured using a Perkin-Elmer Spectrum 100 FT-IR Spectrometer (PerkinElmer, Inc., Waltham, MA USA). Fluorescence spectra were measured on an Agilent Cary Eclipse Fluorescence Spectrophotometer (Agilent Technologies, Inc., Santa Clara, CA, USA). UVVis spectra were measured on an Agilent Cary 60 UV–Vis Spectrophotometer (Agilent Technologies, Inc.). UV light (366 nm) irradiation was carried out with an 8W Three-Way UV lamp (Cole-Parmer Instruments Co., Vernon Hills, IL, USA). Irradiation with visible light (530 nm) was carried out with an Obelux CR9 Forensic Lights Green (Obelux Oy, Helsinki, Finland). A Büchi Melting Point B-540 was used to determine melting points. Toluene was dried over sodium wire. Column chromatographic separation was performed on silica gel 60 (0.063–0.200 mm), using ethyl acetate and hexane mixture as eluent. An analytical TLC (thin layer chromatography) was carried out using Merck precoated silica gel 60 GF-254 with 250 μm thickness plates.

### 2.3. Synthesis

#### 2.3.1. (E)-3-(1-(2,5-dimethylfuran-3-yl)ethylidene)-1-(2-oxo-2H-chromen-6-yl)-4-(propan-2-ylidene) pyrrolidine-2,5-dione (5E)

A mixture of fulgide (1E) (0.115 g, 0.443 mmol) and 6-aminocoumarin hydrochloride (0.105 g, 0.532 mmol) in dry toluene (20 mL) was refluxed under an inert atmosphere with a Dean-Stark adapter for ~70 h. Analytical TLC was used to control the reaction progress. After completion of reaction, solvents were removed by rotavap, the resulting mixture was separated by column chromatography (silica gel, ethyl acetate/n-hexane combination) to give an orange solid (0.045 g, yield: 25%) mp: 170–175 °C. ^1^H NMR (300 MHz, CDCl_3_ , ppm; Figure S1) δ H 7.72 (1H, d, J = 9 Hz), 7.61–7.58 (2H, m), 7.44 (1H, d, J = 9 Hz), 6.47 (1H, d, 9 Hz), 5.95 (1H, s, furyl-H), 2.61 (3H, s, Me), 2.35 (3H, s, Me), 2.26 (3H, s, Me), 2.06 (3H, s, Me), and 1.40 (3H, s, Me). Calculated for C_24_H_21_NO_5_: 403.14; ESI-403.1423 (M+) . FT-IR (ATR, neat) /cm^-1^: 3070, 2923, 2851, 1747, 1724, 1694, 1609, 1570, 1491, 1440.

#### 2.3.2. (E)-3-(dicyclopropylmethylene)-4-(1-(2,5-dimethylfuran-3-yl)ethylidene)-1-(2-oxo-2Hchromen-6-yl) pyrrolidine-2,5-dione (6E)

A mixture of fulgide (2E) (0.2 g, 0.64 mmol) and 6-aminocoumarin hydrochloride (0.15 g, 0.77 mmol) in dry toluene (20 mL) was refluxed under an inert atmosphere with a Dean-Stark adapter for ~70 h. Analytical TLC was used to control the reaction progress. After completion of reaction, solvents were removed by rotavap, the resulting mixture was separated by column chromatography (silica gel, ethyl acetate/n-hexane combination) to give a white-yellow solid (0.33 g, yield: 78%) mp: 214–218 °C, ^1^H NMR (600 MHz, CDCl_3_, ppm; Figure S2): δ H 7.70 (1H, d, J = 9.6 Hz), 7.60–7.59 (2H, m), 7.41 (1 H, d, J = 9 Hz), 6.45 (1H, d, J = 9.6 Hz), 5.91 (1H, s, furyl-H), 3.20 (1H, m, cyclopropyl-H), 2.62 (3H, s, Me), 2.22 (3H, s, Me), 2.13 (3H, s, Me), 0.92 (4H, m, cyclopropyl-H), 0.57 (2H, broad s, cyclopropyl-H), 0.39 (2H, m, cyclopropyl-H), and 0.27 (1H, m, cyclopropyl- H). Calculated for C_28_H_25_NO_5_: 455.5; ESI-456.6 (M+H)^+^. FT-IR (ATR, neat) /cm^-1^: 2922, 2853, 1730, 1708, 1571, 1521, 1492, 1441.

#### 2.3.3. (E)-3-(adamantylidene)-4-(1-(2,5-dimethylfuran-3-yl)ethylidene)-1-(2-oxo-2H-chromen-6-yl) pyrrolidine-2,5-dione (7E)

A mixture of fulgide (3E) (0.156 g, 0.443 mmol) and 6-aminocoumarin hydrochloride (0.105 g, 0.532 mmol) in dry toluene (20 mL) was refluxed under an inert atmosphere with a Dean-Stark adapter for ~70 h. Analytical TLC was used to control the reaction progress. After completion of reaction, solvents were removed by rotavap, the resulting mixture was separated by column chromatography (silica gel, ethyl acetate/n-hexane combination) to give a yellow solid (0.053 g, yield: 24%) mp: 242–247 °C. _1_H NMR (300 MHz, CDCl_3_, ppm; Figure S3) δ H 7.72 (1H, d, J = 9 Hz), 7.64–7.61 (2H, m), 7.43 (1H, d, J = 9 Hz), 5.97 (1H, d, J = 9 Hz), 5.95 (1H, s, furyl-H), 4.43 (1H, broad s, adamantly-H), 2.54 (3H, s, Me), 2.44 (1H, broad s, adamantly-H), 2.26 (3H, s, Me), and 2.09 (3H, s, Me), 1.97–1.77 (12H, m, adamantly-H). Calculated for C_31_H_29_NO_5_: 495.2; ESI-495.237 (M^+^). FT-IR (ATR, neat) /cm^-1^: 3077, 2906, 2846, 1736, 1694, 1614, 1572, 1490, 1443.

#### 2.3.4. (E)-3-(1-(2-methyl-5-phenylthiophen-3-yl)ethylidene)-1-(2-oxo-2H-chromen-6-yl)-4-(pro pan-2-ylidene) pyrrolidine-2,5-dione (8E)

A mixture of fulgide (4E) (0.15 g, 0.443 mmol) and 6-aminocoumarin hydrochloride (0,105 g, 0,532 mmol) in dry toluene (20 mL) was refluxed under an inert atmosphere with a Dean-Stark adapter for ~70 h. Analytical TLC was used to control the reaction progress. After completion of reaction, solvents were removed by rotavap, the resulting mixture was separated by column chromatography (silica gel, ethyl acetate/n-hexane combination) to give a pink solid (0.09 g, yield: 42%). mp: 244–254 °C. ^1^H NMR (300 MHz, CDCl_3_, ppm; Figure S4) δ H 7.73 (1H, d, J = 9 Hz), 7.64-7.60 (2H, m), 7.56–7.53 (2H, m), 7.47–7.39 (3H, m), 7.31 (1H, d, J = 6 Hz), 7.11 (1 H, s, thiophenyl-H), 6.48 (1H, d, J = 9 Hz), 2.70 (3H, s, Me), 2.32 (3H, s, Me), 2.31 (3H, s, Me), 1.37 (3H, s, Me). Calculated for C_29_H_23_NO_4_S: 481.13; ESI-481.1342 (M^+^). FT-IR (ATR, neat) /cm^-1^: 2937, 1754, 1731, 1699, 1628, 1570, 1489, 1439, 1369.

Figure S5 in supporting information shows mass spectra [LC-MS Q-TOF (HRMS)] of 5E, 7E, and 8E.

## 3. Results and discussion

In this project, photochromic fulgides (1E–4E) were synthesized by a multi-step reaction starting from diethyl succinate and other chemical species. The reaction steps consist of the first Stobbe condensation, esterification, the second Stobbe condensation, hydrolysis, and dehydrative cyclization [2,19]. Coumarin-attached new fulgimides (5E–8E) were prepared by refluxing fulgides 1E–4E with 6-aminocoumarin in toluene under an inert atmosphere using the Dean-Stark apparatus (Figure 2).

### 3.1. Photochromism and solvatochromism

**Figure 3:**
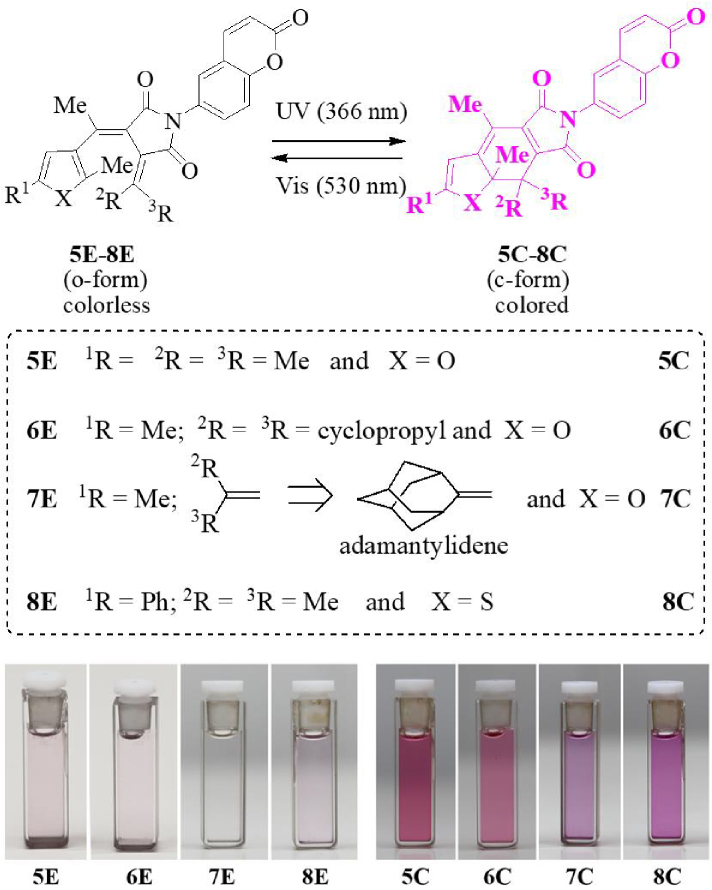
Photoreactions and color change of fulgimides (The photos are from the toluene solutions).

UV (366 nm) and visible (530 nm) lights were used to conduct ring-closure and ring-opening photoreactions of the coumarin-fulgimides (5E–8E) in solution, respectively. Upon UV light irradiation, the coumarin fulgimides showed the color change from colorless (or nearly colorless) to purple because of the formation of highly conjugated c-forms (5C–8C), which can be reverted to the o-forms by the exposure to visible light (Figure 3).

**Figure 4:**
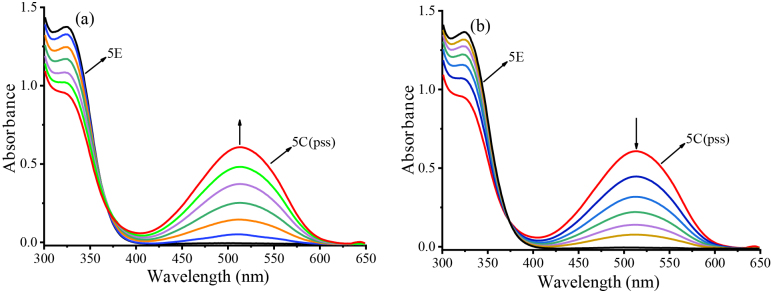
Absorption spectral changes of fulgimide 5E in toluene (1.21 ×10^-1^ M). (a) 5E to 5C (pss) irradiated at 366 nm [0 s, 10 s, 25 s, 1 min, 2 min, 3 min, and 30 s, 8 min (pss)]; (b) 5C (pss) to 5E irradiated at 530 nm (0 s, 5 min , 8 min, 11 min, 15 min, 20 min, 55 min).

For instance, Figure 4 shows UV-Vis absorption spectral changes of fulgimide 5E in toluene. In Figure 4a, 5E (o-form) (1.21 ×10−4 M) absorbs light only in UV region (λmax 324 nm, εmax 11400 mol−1 dm3 cm−1) . When the solution was exposed to 366-nm light at sequential time intervals, a color change from nearly colorless to purple has been observed due to the appearance of a new absorption band in the visible region, which is ascribed to the formation of 5C (c-form). After 8 min of exposure, the new absorption band reached the pss (λmax 513 nm, Absorbance 0.61). Figure 4b shows the reverse photoreaction of 5C to 5E. When the colored solution at pss, which included 5C was exposed to 530-nm light at sequential time intervals, the purple color disappeared gradually, and the initial nearly colorless solution of 5E was regenerated after 55-min irradiation.

Similar forward and reverse photoreactions for 6E, 7E, and 8E in toluene have also been conducted, and UV-Vis spectral data before and after photoreactions are shown in Table 1; and UV-Vis spectral changes before and after photoreactions are shown in Figures S6, S7 and S8 (in supporting information).

Among the fulgimides, the longest absorption band of the c-form in the visible region was observed for 8C, which has the 2-methyl-5-phenylthiophen-3-yl group in place of the 2,5-dimethylfuran-3-yl group of other fulgimides in their o-forms (Figure 2). The reason why 8C has the longest absorption maximum wavelength is explained by the largest conjugation of the c-form owing to the phenyl group on the thiophene ring.

**Figure 5:**
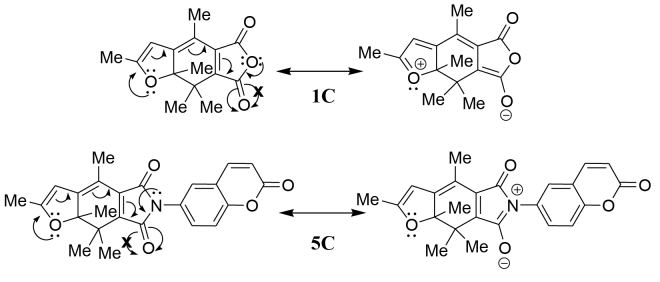
Principal resonance structures of 1C and 5C.

The solvent polarity effects on the absorption spectra of c-forms were investigated. When the photochromic ring closure was carried out in ethyl acetate, the absorption maximum wavelengths of 5C–8C are all blue-shifted compared to the data in toluene. This tendency was reported previously for the fulgide 1C [20]. In more polar acetonitrile, to the contrary, the absorption maximum wavelengths of 5C–7C recovered to the same level of those in toluene, but still much shorter (544 nm) than in toluene (555 nm) in the case of 8C. This tendency is different from the behavior of 1C whose absorption maximum wavelength in acetonitrile (504 nm) is 10-nm longer than that in toluene (494 nm). It is known that the absorption maximum of zwitterionic merocyanine dyes such as the colored form of spiropyrans [21,22] or pyridazinones [23] show a blue shift when the solvent polarity increases. However, the behavior of 5C–8C is not similar to that of merocyanine dyes. Probably the difference of acid anhydride (1C) and imide (5C–8C), and the 2,5-dimethyl-3-furyl (5C–7C) and 2-methyl-5-phenyl-3-thienyl (8C) caused these complex solvent polarity effects. For example, when the resonance structures of 1C and 5C are compared (Figure 5), the longest conjugation of 1C starts from the furan oxygen towards the lower carbonyl group of the acid anhydride. It is the same for 5C. However, because the electronegativity of ether oxygen of the acid anhydride moiety of 1C is larger than that of nitrogen of the imide moiety of 5C, the long conjugation in 1C is not disturbed while that in 5C is disturbed strongly. Thus, the zwitterionic resonance structures of 1C and 5C are different so that the solvent effects of polar solvents on these polar resonance structures become complex.

The UV-Vis absorption spectral data for the coumarin-fulgimides (5E–8E), before and after exposure to the UV light in toluene, ethyl acetate and acetonitrile are presented in Table 1.

**Table 1:** Absorption (UV-Vis) data of coumarin-fulgimides.

Comp.	Solvents		
	Toluene λ_max_/(ε_max_) → λ_max_ /(A)^2)^	Ethyl acetate λ_max_/(ε_max_) → λ_max_/(A)^2)^	Acetonitrile λ_max_/(ε_max_) → λ_max_ /(A)^2)^
5E→5C	324/(11400)→513/(0.61: 7640)	265/(23400)→508 /(0.61: 7200)	263/(17400)→511 /(0.33: 5800)
6E→6C	286/(17300)→530/(0.40: 7030)	256/(28800)→523 /(0.52: 5060)	270/(22400)→530 /(0.49: 5000)
7E→7C	286/(17600)→535/(0.24: 15000)	269/(29200)→529 /(0.30: 14500)	269/(32800)→538 /(0.20: 13100)
8E→8C	297/(39100)→555/(1.11: 6720)	268/(42700)→543 /(1.12: 6950)	270/(28200)→544 /(0.78: 7360)

1) Cell length: 1 cm. Concentration: 5E and 6E: 1.21 ×10^-4^M; 7E and 8E: 1.22 ×10^-4^M. 2) λmax/nm (εmax/mol^-1^dm^3^ cm^-1^) → λmax/nm (absorbance: εmax/mol^-1^dm^3^cm^-1^) at pss.

**Figure 6:**
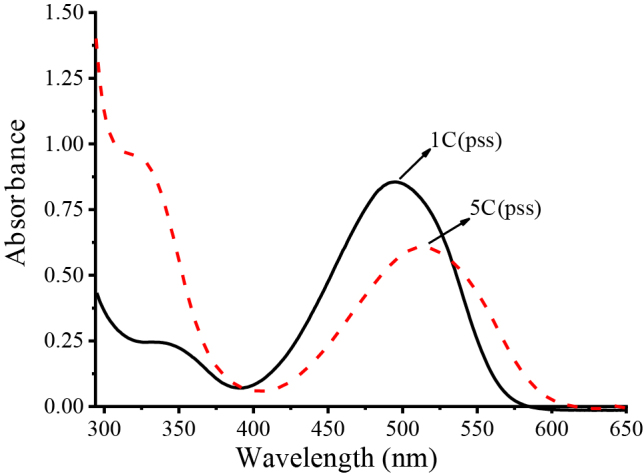
Comparison of c-form of fulgide 1C and c-form of corresponding coumarin-fulgimide 5C [The c-form of fulgide 1C, λmax = 495 nm, and the c-form of fulgimide 5C, λmax = 512 nm at pss in toluene (1.21 ×10^-4^M)].

The starting fulgides are also photochromic, and they can be isomerized to their c-forms by UV irradiation. The conversion of fulgides to their corresponding fulgimides does not only affect the thermal stabilities [9] and fluorescence behaviors [24] but also shifts the absorption maxima (λmax) to longer wavelengths. When the cform of fulgimides are compared with the c-form of starting fulgides, slight bathochromic shifts of the absorption maximum wavelengths (10–20 nm) were observed. For instance: 1C (λmax = 495 nm)→5C (λmax = 512 nm); 2C (λmax = 511 nm)→6C (λmax = 530 nm); 3C (λmax = 520 nm)→7C (λmax = 534 nm), and 4C (λmax = 545 nm)→8C (λmax = 555 nm). A representative example of the comparison of spectra of the corresponding c-forms (1C and 5C) is shown in Figure 6.

Quantum yields for the ring-closure (Φ_E→C_) photoreactions for 5E–8E were determined using the actinometer Aberchrome 540, as described by Heller et al [25]. The quantum yields for coloring at 366 nm in toluene at room temperature were found to be 27% (Φ_5E→5C_), 29% (Φ_6E→6C_), 20% (Φ_7E→7C_), and 19% (Φ_8E→8C_), respectively. Slightly higher quantum yields were observed for 5E and 6E compared with the Aberchrome 540 (Φ_E→C_ = 20%). On the other hand, the lowest quantum yield was recorded for 8E. The reason of lower quantum yield could be explained by the larger aromatic stabilization energy [26] of the thiophene ring (77.6 kJ/mol) compared to that of the furan ring (61.7 kJ/mol) on other fulgimides. During the photoreaction aromatic rings on the fulgimides lose their aromaticity to give more conjugated closed-ring forms. As the result, furylfulgimides (5E–7E) turned into their c-forms slightly more efficiently than the thienylfulgimide (8E).

### 3.2. Fluorescence property

**Figure 7:**
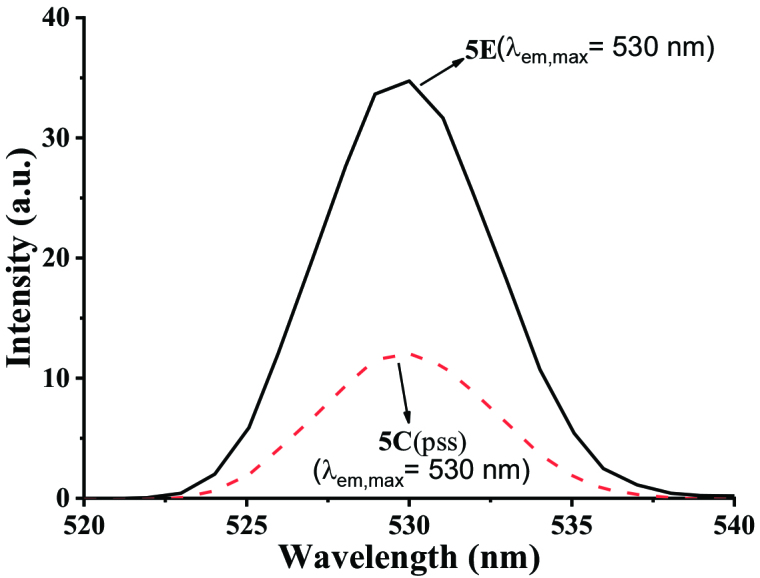
Fluorescence emission spectral changes in toluene (1.21 ×10^-4^M) (λex = 350 nm) 5E to 5C (pss) irradiated at 366 nm.

Since the coumarin derivatives [17] are known to be fluorescent, it is natural that the coumarin attached fulgimides can also be fluorescent, and the fluorescence properties may be switched (on/off) by the photochromic transformation. The fluorescence properties of 5E–8E (o-forms) were examined in polar and nonpolar solvents at room temperature. When they are excited with 350-nm light, intense emission bands of 5E–8E were observed at 530 nm in toluene, and around 541 nm in ethyl acetate and acetonitrile. After the coumarin-attached fulgimides were irradiated with the UV light until they reach pss, their fluorescence spectra were measured again. Florescence data of o-forms and c-forms are listed in Table 2. A representative fluorescence spectral change, before and after the UV irradiation of 5E in toluene, is presented in Figure 7. Similar spectral changes for the 6E, 7E, and 8E are given in Figures S9, S10 and S11 (in supporting information). As can be seen in the spectrum, the o-form has a broad and intense emission band (λem, max = 530 nm), while the pss solution shows relatively weaker emission band at the same wavelength. The emissions of coumarin-attached fulgimides in their pss stages in toluene were quenched by 66%, 47%, 44% and 91%, compared with their o-forms (5E–8E), respectively. A similar behavior was also observed in ethyl acetate and acetonitrile. For instance, the emission of 8E in ethyl acetate was quenched by 95% at the UV-pss. Except few examples [13], an open ring isomer of fluorescence-switchable fulgide derivative exhibits an intense fluorescence emission band in the visible region, while the fluorescence of its UV-pss solution is quenched significantly [16,18,27].

**Table 2:** Fluorescence data of coumarin-fulgimides.

Comp.	Solvents		
	Toluene λ_em_/nm (rel. int) → λ_em_/nm (rel.int)	Ethyl acetate λ_em_/nm(rel.int) → λ_em_/nm (rel.int)	Acetonitrile λ_em_/nm(rel.int) → λ_em_/nm (rel.int)
5E→5C	530 (1)→530 (0.34)	541 (1)→541 (0.30)	541 (1)→541 (0.53)
6E→6C	530 (1)→530 (0.53)	541 (1)→541 (0.15)	541 (1)→541 (0.19)
7E→7C	530 (1)→530 (0.56)	542 (1)→541 (0.47)	542 (1)→541 (0.68)
8E→8C	530 (1)→530 (0.09)	540 (1)→541 (0.05)	540 (1)→541 (0.30)

Concentration: 5E and 6E: 1.21 ×10^-4^M; 7E and 8E: 1.22 ×10^-4^ M. Excitation wavelength: 350 nm.

**Figure 8:**
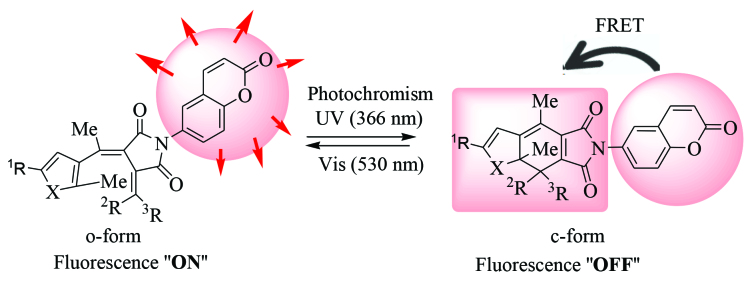
Fluorescence resonance energy transfer (FRET) in the closed-ring form of coumarin-fulgimides.

The reason why the o-forms 5E–8E are fluorescent and the c-forms 5C–8C are nonfluorescent could be explained by the occurrence of Förster resonance energy transfer (FRET) from the coumarin part to the fulgimide unit in the c-form (Figures 1 and 8).

Based on the hypothesis that only o-form of the coumarin-attached fulgimides are fluorescent and c-forms are not fluorescent, the molar absorption coefficients of the absorption bands in the visible region of c-forms will be determined since the quenched fluorescence at the pss compared to the o-form is due to the generation of c-form. Thus, the molar absorption coefficients of c-forms in the visible region calculated in this way are shown in Table 1.

## 4. Conclusions

Four new photochromic as well as florescent coumarin-attached fulgimides have been synthesized from the corresponding fulgides. Although their open-ring forms are fluorescent, the fluorescence at the UV-pss state is substantially quenched because the closed-ring forms are not fluorescent. The reason of the quenching of the fluorescence was explained by FRET type energy transfer. The result indicates that the fulgimides synthesized in this research are suitable for fluorescence-switching molecules by their photochromism.

Supplementary Materials
