# Supplementary material for: Photochromic and fluorescence properties of coumarin fulgimides
Source: Turk J Chem. 2020 Aug 18;44(4):1031–42. doi: 10.3906/kim-2003-31 (PMC7751943; doi:10.3906/kim-2003-31)
Supplement: Supplementary file 1 — Supplementary Materials [file turkjchem-44-1031-sup001.pdf]

Supporting Information

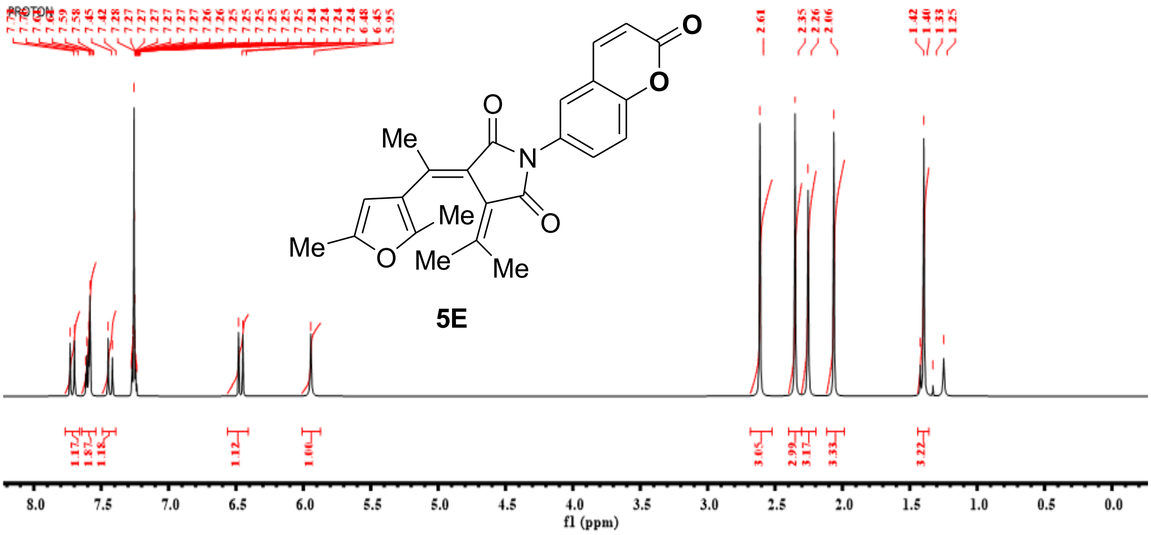

Figure S1. ^{1}H NMR Spectra (300 MHz, $CDCl_3$) of **5E**.

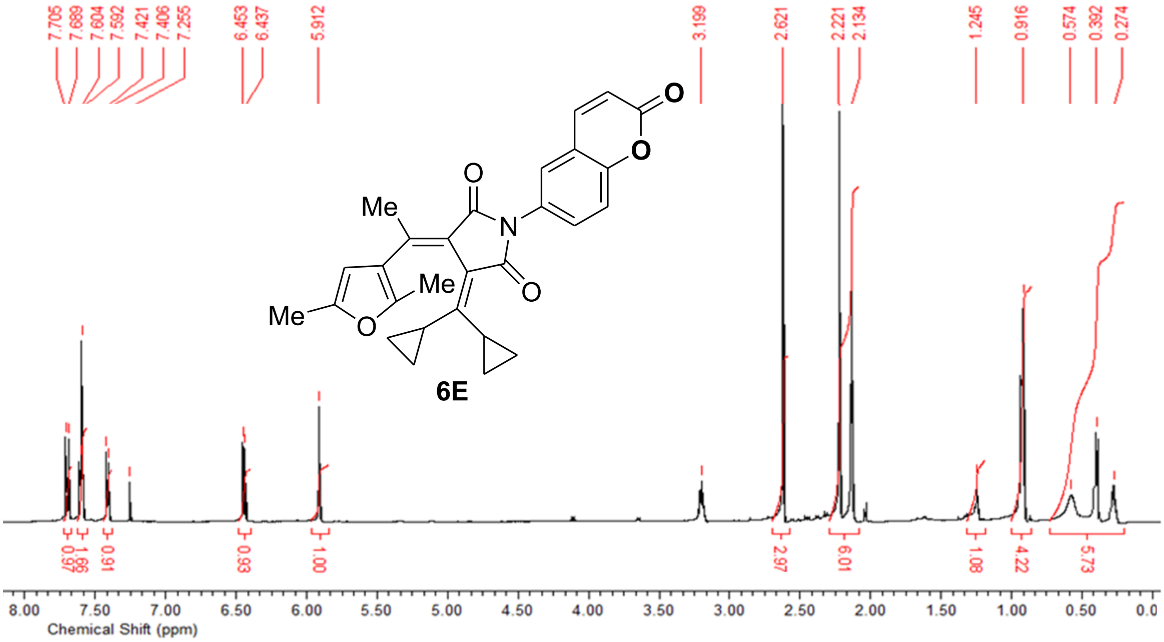

Figure S2. ^{1}H NMR Spectra (600 MHz, $CDCl_3$) of **6E**.

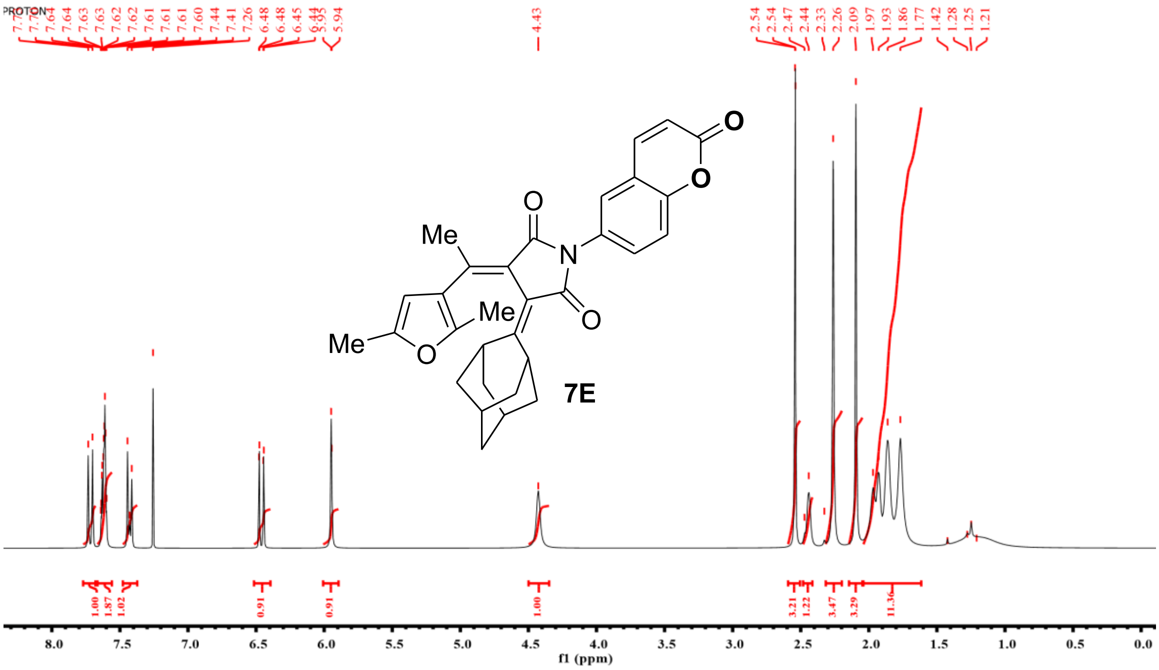

Figure S3. ^{1}H NMR Spectra (300 MHz, $CDCl_3$) of **7E**.

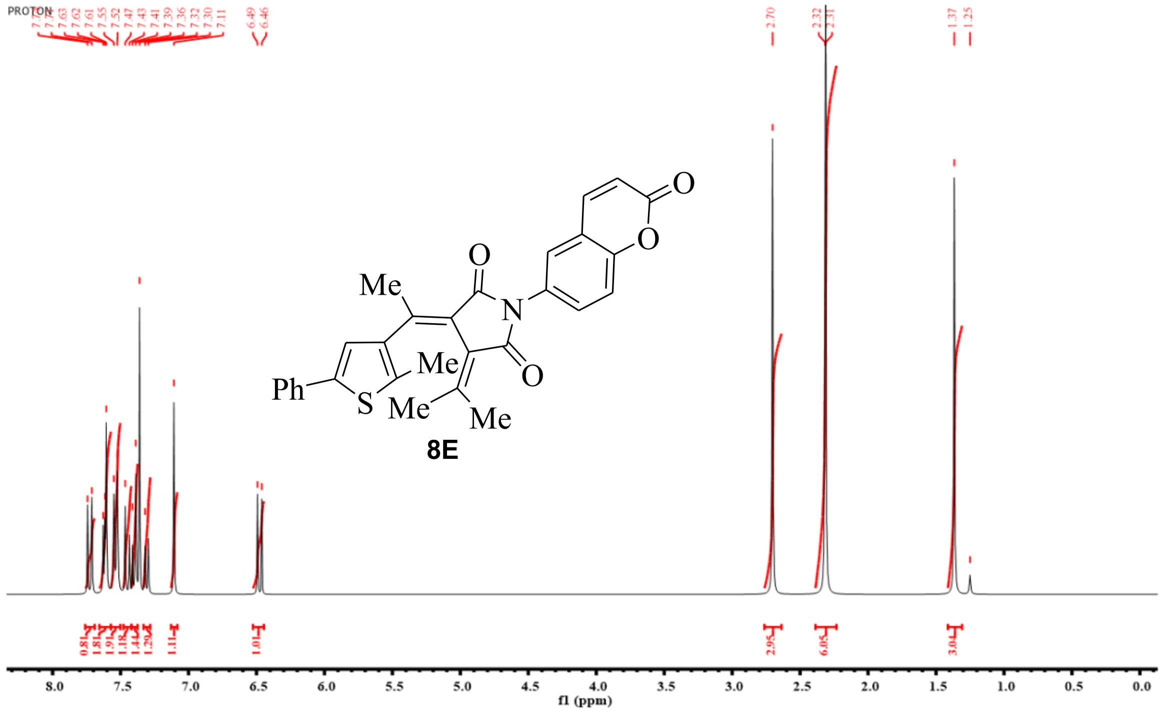

Figure S4. ^{1}H NMR Spectra (300 MHz, $CDCl_3$) of **8E**.

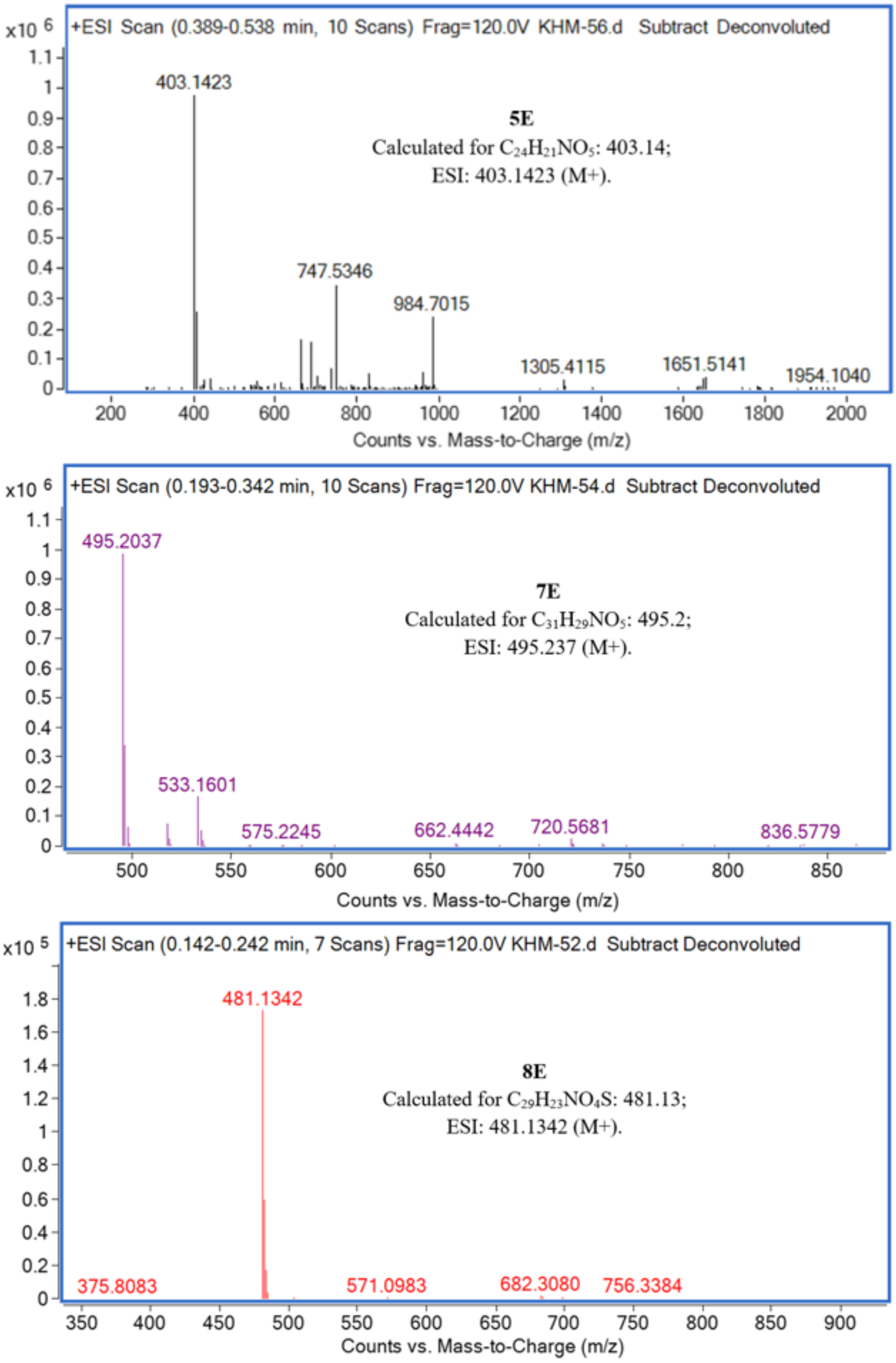

Figure S5. Mass Spectra (LC-MS Q-TOF (HRMS)) of **5E, 7E,** and **8E**.

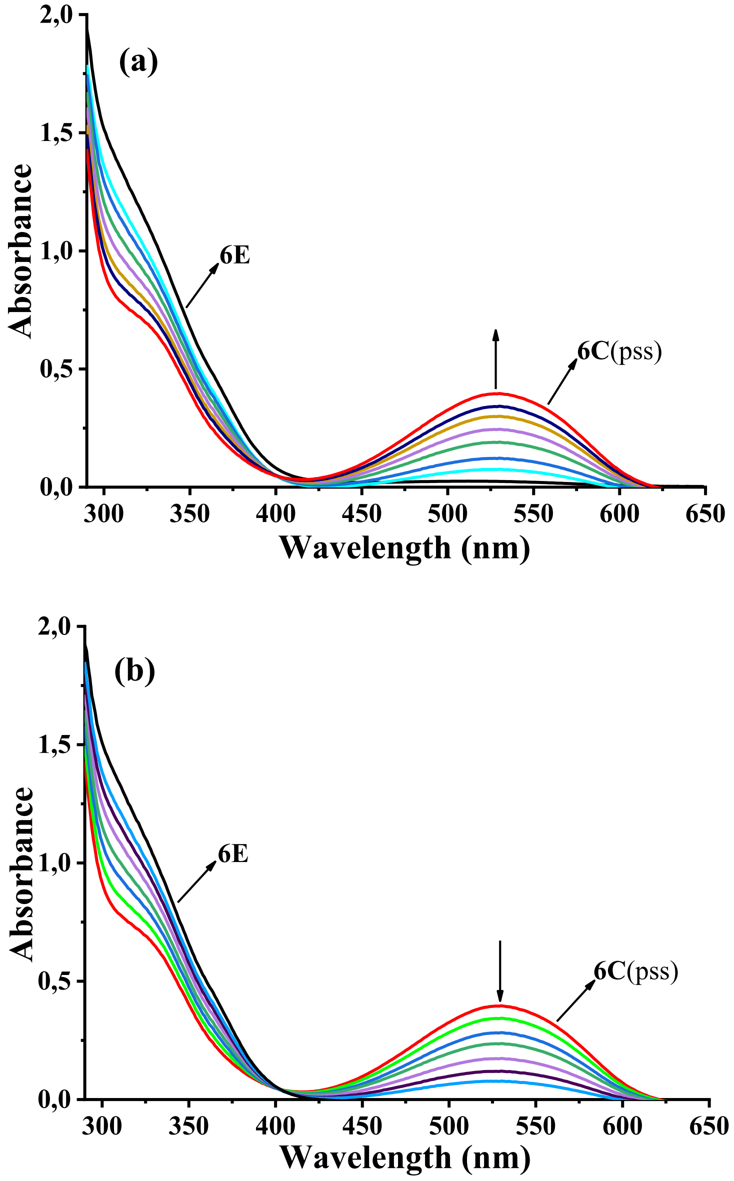

Figure S6. Photoreaction and absorption spectral changes in toluene (1.21 X 10^{-4} M). (a) **6E** to **6C** (pss) irradiated at 366 nm (0 sec, 15 sec, 25 sec, 40 sec, 1 min,1 min and 30 sec, 2 min and 10 sec, 5 min and 40 sec.); (b) **6C** (pss) to **6E** irradiated at 530 nm (0 sec, 10 sec, 40 sec,1 min, 1 min and 30 sec, 2 min, 2 min and 30 sec, 5 min and 30 sec.).

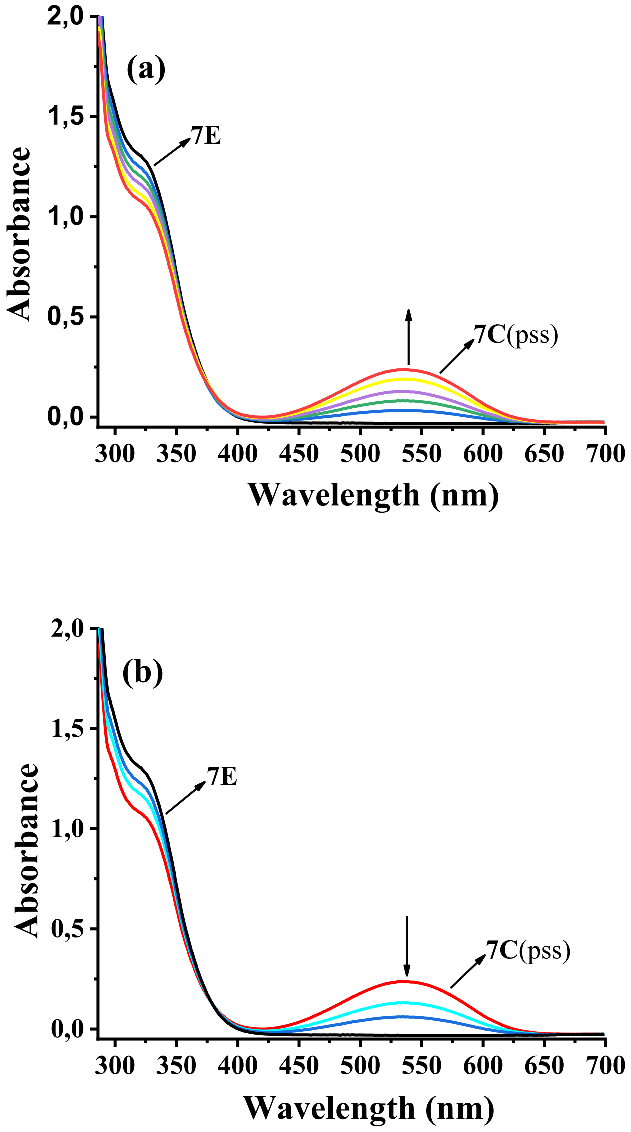

Figure S7. Photoreaction and absorption spectral changes in toluene (1.22 X 10^{-4} M). (a) **7E** to **7C** (pss) irradiated at 366 nm (0 sec, 25 sec, 55 sec, 1 min and 35 sec, 3 min and 25 sec, 7 min and 50 sec); (b) **7C** (pss) to **7E** irradiated at 530 nm (0 sec, 10 sec, 25 sec, 3 min).

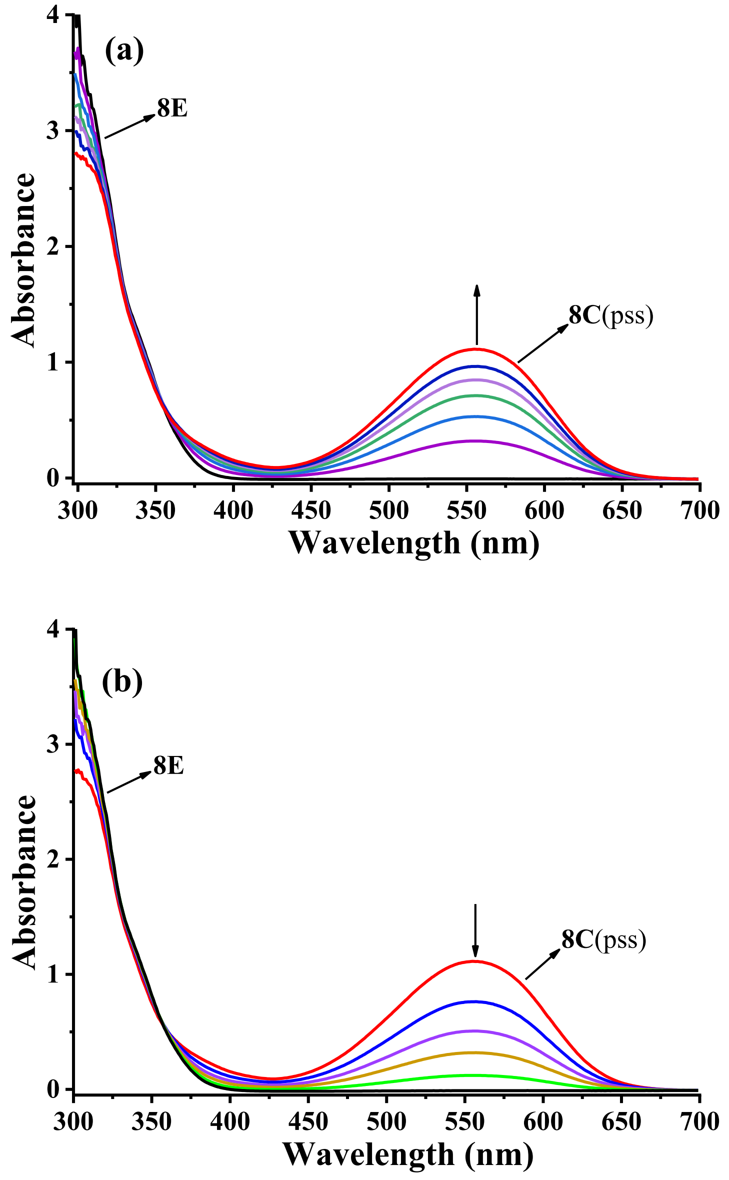

Figure S8. Absorption spectral changes of fulgimide **8E** in toluene (1.22 X 10^{-4} M) (a) **8E** to **8C** (pss) irradiated at 366 nm (0 sec, 1 min, 2 min, 3 min and 30 sec, 5 min and 30 sec, 8 min and 30 sec, 28 min (pss)); (b) **8C** (pss) to **8E** irradiated at 530 nm (0 sec, 4 min, 5 min and 30 sec, 7 min, 9 min, 20 min).

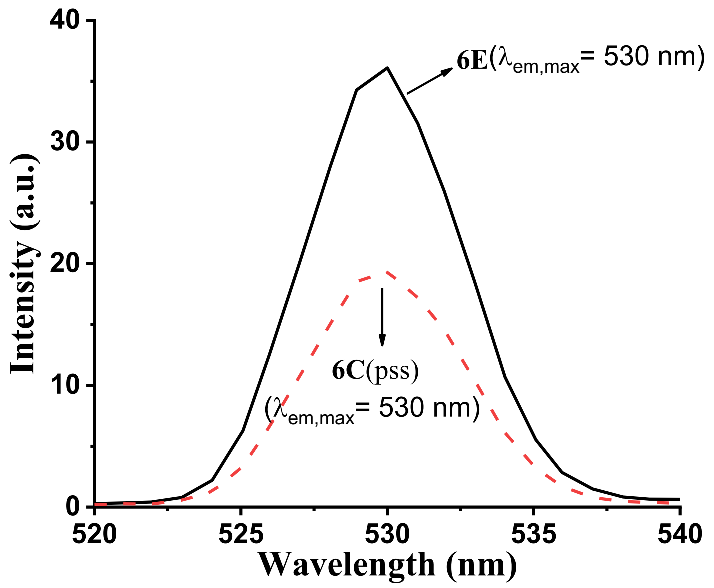

Figure S9. Fluorescence emission spectral changes in toluene (1.21 X 10^{-4} M) (λex = 350 nm), **6E** to **6C** (pss) irradiated at 366 nm.

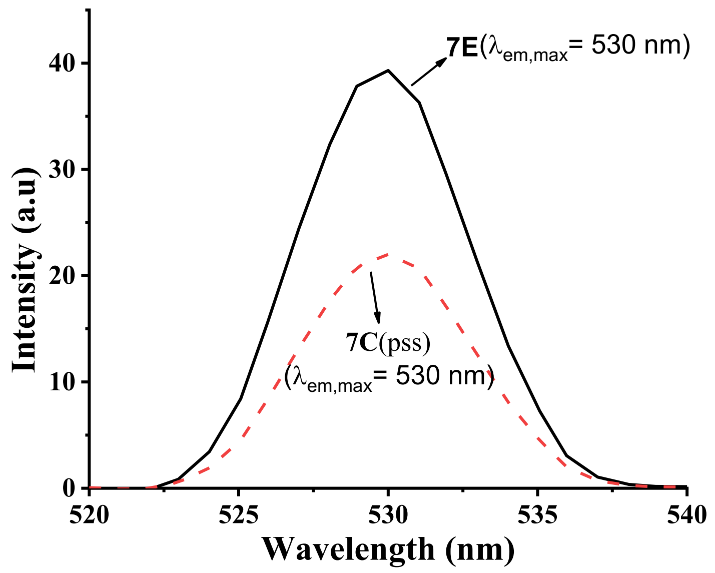

Figure S10. Fluorescence emission spectral changes in toluene (1.22 X 10^{-4} M) (λex = 350 nm), **7E** to **7C** (pss) irradiated at 366 nm.

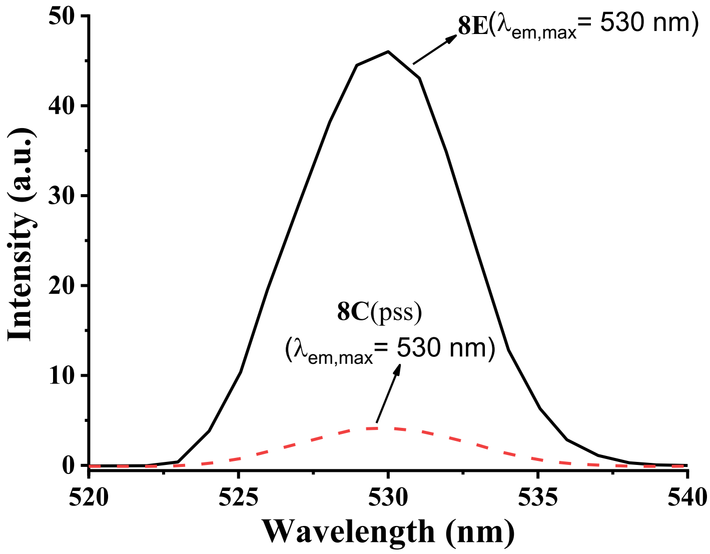

Figure S11. Fluorescence emission spectral changes in toluene (1.22 X 10^{-4} M) (λex = 350 nm), **8E** to **8C** (pss) irradiated at 366 nm.
